# Supplementary material for: Bystanders of intimate partner violence against women and their willingness to intervene: An analysis of secondary data in Spain (2005–2020)
Source: PLoS One. 2022 Sep 20;17(9):e0274822. doi: 10.1371/journal.pone.0274822 (PMC9488790; doi:10.1371/journal.pone.0274822)
Supplement: Attachment — Submitted filename: Response_to_Reviewers.docx [file pone.0274822.s001.docx]

Dear editor,

According to the editor and reviewers’ suggestions, we have revised and changed our paper “Bystanders of intimate partner violence against women and their willingness to intervene: an analysis of secondary data in Spain (2005-2020)" (PONE-D-22-02976) in order to improve it. Specifically we have made the following changes:

**Journal requirements:**

**1. Please ensure that your manuscript meets PLOS ONE's style requirements, including those for file naming.**

**Author’s response**: We have ensured that our manuscript meets PLOS ONE's style requirements, including those for file naming. Specifically, we have followed the PLOS ONE style templates (https://journals.plos.org/plosone/s/file?id=wjVg/PLOSOne_formatting_sample_main_body.pdf and https://journals.plos.org/plosone/s/file?id=ba62/PLOSOne_formatting_sample_title_authors_affiliations.pdf) and the rules related to tables (https://journals.plos.org/plosone/s/tables) and figures (https://journals.plos.org/plosone/s/figures).

It should be noted that in this case (changes in document format or in Tables format) we have not used the tool "track changes" option in Microsoft Word for avoiding mistakes and too much coloured text.

In the case of the numbered list of references**, the numbering has been modified to adapt it to the current references**, presenting only the correct numbering (the original numbering has not been kept for clarity). To make them easier to identify, the new references introduced in response to the reviewers' suggestions have been marked as an insertion.

**2. In your Data Availability statement, you have not specified where the minimal data set underlying the results described in your manuscript can be found**.

**Author’s response**: The methodological strategy used was an analysis of secondary data obtained and processed by other sources. In this case, quantitative data taken from sociological surveys conducted in Spain by different institutions and administrative data related to the records of complaints for intimate partner violence against women and they are fully available on the web freely for anyone who wishes to consult them.

The URLs from the Centro de Investigaciones Sociologicas (<https://www.cis.es/cis/opencms/EN/index.html>) where the quantitative data from sociological surveys are available are:

- Social perception of gender violence (study 2968): <https://www.cis.es/cis/opencm/ES/1_encuestas/estudios/ver.jsp?estudio=14086>

- Social perception of gender violence among adolescents and youth (study 2992): <https://www.cis.es/cis/opencm/ES/1_encuestas/estudios/ver.jsp?estudio=14106>

- Macro-survey on violence against women 2014 (study 3027): <https://www.cis.es/cis/opencm/ES/1_encuestas/estudios/listaMuestras.jsp?estudio=14084>

- Macro-survey on violence against women 2019 (study 3235): <https://www.cis.es/cis/opencm/ES/1_encuestas/estudios/listaMuestras.jsp?estudio=14470>

The URL from the Statistics Portal of the Spanish Government Delegation for Gender Violence where the administrative data related to the records of complaints for intimate partner violence against women are available are: <http://estadisticasviolenciagenero.igualdad.mpr.gob.es/>

All these data are fully anonymized.

**3. We note that you have stated that you will provide repository information for your data at acceptance.**

**Author’s response:** Apologies for the mistake.

All data files are available from the Centro de Investigaciones Sociologicas (<https://www.cis.es/cis/opencms/EN/index.html>) and from the Statistics Portal of the Spanish Government Delegation for Gender Violence (<http://estadisticasviolenciagenero.igualdad.mpr.gob.es/>)

**4. Please include a copy of Table 11 which you refer to in your text on page 23.**

**Author’s response:** Apologies for the mistake, which has been corrected in the text.

On line 654 it said "see Table 11", when it should have said "see Table 2" (now, with changes introduced it says “see Table 5”).

**5. We note you have included a table to which you do not refer in the text of your manuscript. Please ensure that you refer to Table 2 in your text; if accepted, production will need this reference to link the reader to the Table.**

**Author’s response:** Apologies for the mistake, which has been corrected in the text.

On line 654 it said "see Table 11", when it should have said "see Table 2" (now, with changes introduced it says “see Table 5”).

**Financial disclosure.**

**Author’s response:** According to the instructions of Spanish Minister, the disclosure sentence may be:

This work was financed by the Spanish State Research Agency (Agencia Estatal de Investigación), through the Research Project PID2019-104006RB-I00/MCIN/AEI/10.13039/501100011033

**Additional Editor Comments**

**This paper addresses a matter of great interest: the prevention of violence against women in intimate partner relationships knowing the behavior and willingness to intervene of bystanders. The approach of the study, getting valuable information regarding IPVAW from macro-surveys, enriches the knowledge in this field.**

**However, the manuscript presents important limitations that should be revised and amended. The paper advances knowledge very little, as it synthesizes what is already described in the reports of the surveys, without providing a theoretical analysis and hypotheses based on previous scientific literature. A deeper discussion based on existing knowledge is needed to make a real good contribution to this field of knowledge.**

**Authors' reply:** We have taken all the reviewers' suggestions into account and have carried out a major revision of the paper.

**The Introduction section lacks a complete revision of previous research. As pointed by reviewer 2, this section must show what we know about this topic, what is consistent and inconsistent in previous research, and how the current study contributes to addressing these gaps. The article does not have a strong theoretical foundation and lacks specific hypotheses (it is not well justified why it is not necessary to state hypotheses). The frame of the study must be elaborated and this whole section redone.**

**Authors' reply:** We have taken all the reviewers' suggestions into account and have carried out a major revision of the introduction section. The specific response to the reviewer indicates the changes made and where they are placed in the manuscript.

Due to the multiple secondary studies used, the paper needs a reorganization of the information, which should be organized and synthetized in a clearer way (reviewer 2 recommends a summary table, what may be a good idea).

**Authors' reply:** We have taken all the reviewers' suggestions into account and have carried out a major revision of the method section (including a summary table of the secondary studies used). The specific response to the reviewer indicates the changes made and where they are placed in the manuscript.

**The paper presents too many figures, so authors should revise them and reorganize the information.**

**Authors' reply:** We have taken all the reviewers' suggestions into account and reduced the figures (from 14 to 9) and reorganized the information.

**In addition, as reviewers agree, further information about the variables, the question format, etc., should be provided.**

**Authors' reply:** We have taken all the reviewers' suggestions into account and have carried out a major revision of the method section (including a table with the variables analysed). The specific response to the reviewer indicates the changes made and where they are placed in the manuscript.

**The discussion section must be deeply revised. No strong interpretations of results are given based on existing knowledge in this field. Therefore, this section should consider and be based on the revised Introduction section that must be done.**

**Authors' reply:** We have taken all the reviewers' suggestions into account and have carried out a major revision of the discussion section. The specific response to the reviewer indicates the changes made and where they are placed in the manuscript.

**Referees requirements:**

**Referee 1:**

**The manuscript entitled 'Bystanders of intimate partner violence against women and their willingness to intervene: an analysis of secondary data in Spain (2005-2020)' deals with the issue of prevention of violence against woman in intimate partner relationships taking into account the immediate environment of the victim, through the analysis of data obtained in various surveys conducted in Spain over 15 years. Given that encouraging the helping behavior of the victim's environment is a prevention strategy that has been considered essential, knowing the behavior and willingness to intervene of the bystanders is of great interest. However, there are some important limitations to note in the manuscript.**

**Author’s response**: As reviewer 1 points out, “Given that *encouraging the helping behavior of the victim's environment is a prevention strategy that has been considered essential, knowing the behavior and willingness to intervene of the bystanders is of great interest*”.

That is precisely why, as we pointed out in the summary of the article (lines 28-30; lines 199-202), the objective of our study is **to estimate the volume of bystanders in cases of IPVAW** in Spain between 2005 and 2020 (since the entry into force of Organic Law 1/2004 to the present), their willingness to intervene and, in the case of intervention, the type of helping behavior (real or hypothetical) preferred by them. This objective leads us to focus on whether there are witnesses in cases of violence against women (VAW) in Spain and what we know about their behaviour. This research is therefore a preliminary step towards understanding the state of the art (are there VAW witnesses in Spain? How do they behave?), and, on this basis, to be able to design more effective intervention programs to prevent VAW in the future.

A scoping review on bystander helping behaviours in VAW cases in Spain showed us that there was little previous research on this topic in Spain, and, consequently, little information. Precisely, one of the sources of information available and reliable were some sociological surveys that show how, although VAW is often seen as a hidden problem, there are in fact bystanders to this violence. That is why this study uses these sources: survey data (as the surveys of social perception of gender violence and the 2014 and 2019 macro-surveys), and administrative data (as the database of reports filed).

In summary, it is important to note that the objective of our study is not to “*deal[s] with the issue of prevention of violence against woman in intimate partner relationships taking into account the immediate environment of the victim*”; rather it is to reflect on the bystander behavior and their willingness to intervene in cases of VAW in Spain.

Related to this, we have improved the description of the objective of the study in order to better identify it and avoid any possible confusion in this regard (see lines 199-212).

**First, the introduction does not provide an adequate background of previous scientific literature. In fact, what the authors do is a very detailed description of the legislative framework and the surveys carried out in Spain in a theoretical vacuum and a marked absence of previous literature on the subject. In short, they do not present an adequate 'state of the art' to understand the results of the study or how this study advances existing knowledge in this field. There are a significant number of previous studies in this area that are not mentioned or taken into account (e.g., Felson & Paré, 2005; Gracia & Herrero, 2006; Kim & Ferraresso, 2021; Lazarus & Signal, 2013). This section should be redone, framing the study appropriately and presenting the legislative and survey description in Spain in a much shorter way, in a sub-section reflecting the 'present study'.**

**Author’s response**: Thank you to the reviewer 1 for their suggestions. In fact, as reviewer 1 points out, the introduction did not present an adequate 'state of the art' to understand the existing knowledge in the field of bystander behaviour in cases of VAW in Spain.

Therefore, taking into account the recommendation of the reviewer 1, the following has been done:

- The explanation about the VAW legislative situation in Spain has been reduced (we have eliminated the lines 85-111).

- The detailed explanation about sociological surveys in Spain has been removed (we have eliminated the lines 112-172). This explanation has been moved to the method section and it is described following the recommendations of reviewer 2.

- A description on the state of the art regarding the object of study (the witnesses in VAW cases in Spain and their behaviour) has been added (see lines 173-193).

We believe that all these changes now provide an adequate background of previous scientific literature on the topic.

**In the design section, the authors indicate that they will use a descriptive and associative analysis strategy. They also point out that for descriptive analyses it is not necessary to state hypotheses, but what happens with associative analyses? Based on previous scientific literature, the type of relationships expected to be found between variables and in which direction should be proposed.**

**Author’s response**: According to the reviewer suggestion, we have added the relationships expected to be found between variables (see lines 231-234).

**A more detailed description of how variables are measured should be given in the variables section.**

**Author’s response**: According to the reviewers 1 and 2 suggestion, we have included a new table (Table 2, see line 309) with all this information about each the variables analysed (the variable, the question, and the question format).

**In general, the results described in the paper advance knowledge very little, as they lack a theoretical analysis and hypotheses based on previous scientific literature, beyond synthesizing what is already described in the reports of the surveys themselves and which the reader can find directly in these sources of information. Although some comparative analyses are made according to sex and age, no deeper interpretations of these results are given based on existing knowledge in this field.**

**Author’s response:** According to the reviewer’s 1 and 3 suggestions, we have remarked the evolution of the estimates on the volume of bystanders in Spain across last years (including the results of this paper) (lines 675-703). Certainly, some of the surveys analyzed are old, but the results they provide offer more complete information on the subject. Moreover, the 4 sociological surveys provide additional valuable information on the (hypothetical) responses of witnesses in IPVAW cases, which offer us the most important result of this work: there is a significant gap between what witnesses say they will do and what they actually do (as shown by the data on complaints). Certainly, social desirability is one of the problems we encounter, but not the only one. We understand that the main result of this paper is precisely to highlight these contradictions between what is said will be done and what is actually done and stated some of the possible explanations, opening the way for its in-depth study in future works.

**Felson, R. B., & Paré, P. P. (2005). The reporting of domestic violence and sexual assault by nonstrangers to the police. Journal of Marriage and Family, 67(3), 597-610.**

**Gracia, E., & Herrero, J. (2006). Public attitudes toward reporting partner violence against women and reporting behavior. Journal of Marriage and Family, 68(3), 759-768.**

**Kim, C., & Ferraresso, R. (2021). Factors associated with willingness to report intimate partner violence (IPV) to police in South Korea. Journal of Interpersonal Violence, Doi: 0886260521990837.**

**Lazarus, K., & Signal, T. (2013). Who will help in situations of intimate partner violence: Exploring personal attitudes and bystander behaviours. International Journal of Criminology and Sociology, 2, 199-209.**

**Auhor’s response:** Thank you for the suggestions. We have added all these references.

**Referee 2:**

**Introduction:**

**They, however, do not appropriately discuss previous research (e.g., what do we know about this topic, what is consistent and inconsistent in previous research, and how the current study contributes to addressing these gaps). I think this is particularly important when it comes to justifying the selection of age and gender as key variables for the analysis (instead of education, income, or any other variable).**

**Auhor’s response**: According to the reviewer suggestion, we have included a discussion about the previous research on the topic and the main results obtained (see lines 173-193).

**The article at the moment does not have a strong theoretical foundation and lacks specific hypotheses. I encourage the authors to review the front-end of their manuscript to make it more theoretically driven and better justify their methodological decisions.**

**Auhor’s response**: According to the reviewer suggestion, we have added specific hypothesis and a theoretical foundation related to the relevance of bystander behaviour to improve the IPVAW prevention (see lines 64-65 and lines 231-234).

**In addition to this, please review this section to fix some typos/grammatical issues and amend the acronym used when referring to the Centro de Investigaciones Sociológicas (as shown on their website, they maintain their Spanish name and acronym in English:** <https://www.cis.es/cis/opencms/EN/8_cis/quienessomos/>).

**Author’s response**: According to the reviewer suggestion, we have revised the all section and also we have amended the acronym used to the Centro de Investigaciones Sociológicas (CIS) in all the text.

**2) Methods:**

**This section is a bit hard to follow at the moment given the multiple secondary studies used. I would recommend creating a summary table that provides information for each study (organism in charge of data collection, population of interest, data collection dates, language(s) in which the survey was administered, mode(s) of data collection, achieved sample size, response rates, and any other relevant information).**

**Author’s response**: According to the reviewer suggestion, we have included a new table (Table 1, see line 243) with all this information about the four sociological surveys analysed.

**I would like to see a similar table with the question wording for each of the variables, and further information about the question format (at the moment it is hard to know whether some questions were closed- or open-ended, if they were exclusive or mark-all-that-apply, but these are the main variables of the study and clarity is paramount here).**

**Author’s response**: According to the reviewer suggestion, we have included a new table (Table 2, see line 309) with all this information about each the variables analysed (the variable, the question, and the question format).

**I am also concerned that there is no mention, in the "Data Analysis" section to the weights used in the analysis to account for the sampling design and nonresponse. To my knowledge, the CIS provides them and they should be used to generate figures/conduct analysis. Could the authors confirm that they used the weights, and if not, update their analyses to incorporate them when available?**

**Author’s response**: Effectively, the CIS surveys provide some weighting weights for the representativeness of cases in the sample. We have included this information in Table 1, which describes the characteristics of the sociological surveys analysed.

It is important to note that including these weights for analysing the data in our article is not appropriate for the following reasons:

- The fact sheets for studies No. 2968 and No. 2992 provided by the CIS state "no weighting applicable".
- The fact sheets for studies no. 3027 (2014) and no. 3235 (2019) provide two weights for two types of data treatment: 1) weights "to treat the sample as a whole" and 2) weights "to make estimates for the Autonomous Communities". Neither of these two assumptions applies to our article. Our analyses of these data are carried out descriptively on a sub-sample (women who reported having suffered some type of gender-based violence). But no inference is made about the general population (case 1), nor about differences between autonomous communities (case 2), for which it would be necessary to use representativeness weights as pointed out by reviewer 2.

**I would also encourage the authors to indicate the software that they used to conduct the analyses, as this is best practice for replicability.**

**Author response**: According to the reviewer suggestion, we have added the software used to conduct the analyses (see line 330-331).

**With regards to the files, I do not think that providing combined data for a 10-year period, and then separate data for 2020 is the best approach. I would report the data by year to identify any relevant patterns over time rather than combining the first 10 years. This would not only provide more granular information, but would also help to assess whether changes with respect to 2020 reflect previous patterns or point to something else.**

**Author response:** According to the reviewer suggestion, we have included in a Table (see Table 5) the data about reports filed by year from 2009 to 2020 (see line 658) and we have analysed all data combined for having a whole comprehension and to try to identify some pattern (see lines 635-657).

**3) Results:**

**Were any corrections used to account for the multiple comparisons? Because of the many bivariate tests that you conduct, the risk of Type I error is high and it would be helpful to describe how this has been minimised. I would also like to see effect sizes in addition to p-values to better assess the magnitude of the differences. With X2, for example, you can use Cramer´s V. I also suggest proofreading this section to amend some typos.**

**Author’s response:** According to the reviewer suggestion, we have incorporated Cramer's V to give an idea of the size of the effect.

Regarding the accumulation of type I error due to multiple comparisons with the same data, the Bonferroni correction was applied where appropriate. The p-values have been modified in the text with this adjustment. In those cases where the SPSS program provides a p-value = .000, it has been maintained in the text as "p < 0.001" since the exact p-value is not available and, obviously, any correction applied would still be significant at that value.

**Currently there are too many figures. I would encourage the authors to consolidate their figures and find ways to combine the information. The results section is also a bit repetitive as the findings are disaggregated by sex, by age, and then by sex and age. In terms of the figures, I think they can be made more self-explanatory with a few tweaks. For example, it is unclear to me what the arrows represent and I could not find an explanation on the Figures themselves. The sample size should be specified, and the bars should be accompanied by confidence intervals, to reflect the uncertainty around the estimates.**

**Author’s response:** According to the reviewer suggestions, we have made the following changes:

- We have reduced the number of figures (from 14 to 9). We have removed Figure 6 because it provided information that could easily be described in the text. Figures 11, 12, 13 and 14 have been removed because they were unclear. The information in figures 11 and 12 has been included in Table 3, the information in figure 13 has been included in Table 4, and the information in figure 14 has been included in Table 5.
- It was decided to keep figures 1 to 5 and 7 to 10 as they provide essential information for the purpose of the study and it was unclear to include the information they contain in the text.
- We have also included the respective 95% confidence intervals in all figures. Given the doubts expressed by the reviewer, and the additional information provided by the confidence intervals, we have decided to remove the arrows because it could cause some confusion (rather than adding clarity).
- We have included the sample size in each case for a better comprehension of the results obtained.

**In the case of Figures 12 and 14, readability is quite poor, given that there is little differentiation across categories. I encourage the authors to explore other alternatives to present their data.**

**Author’s response:** According to the reviewer suggestions, the information in figure 12 has been included in Table 3, and the information in figure 14 has been included in Table 5.

**With the youth study (2992), what is the rationale for the age grouping? 15-17 seems much more restricted that 18-29. Were the groups balanced in terms of sample size? Perhaps creating further groups (18-20, 21-23...) would be preferred here.**

**Author’s response**: The reason for creating these two age groups is to be able to compare the results of the 2012 survey with the adult population and the 2013 survey with the young population. In this way, in both cases we had a group of 18-29 year olds and we could make comparisons between the results obtained in both studies.

**When presenting the findings from the macro surveys, because you´re using sub-samples (e.g., only women who reported IPV, only women in current relationships) it is essential to provide the sample sizes in all cases. As currently worded, reporting percentages only, it is not always possible for readers to see how many cases were used to compute the figures provided.**

**Author’s response**: According to the reviewer suggestions, we have included the sample size in each case for a better comprehension of the results obtained.

**As shown in Table 1, some of the cells have very few cases (<20). Because of this, I would encourage the authors to be more cautious when interpreting the findings, reminding readers that some of the findings are based on very small sample sizes. This would be something helpful to discuss among the limitations of the study.**

**Author’s response**: According to the reviewer suggestions, we have added this limitation of our study in the in the discussion section.

**As I suggested earlier, I would report the data for each year in Table 2, so that readers have access to all of the information and can better assess trends, rather than getting only three data points and assuming linearity in between.**

**Author’s response**: According to the reviewer suggestions, the information in figure 14 has been included in Table 5 (before the changes, figure 2). So, the data about reports filed by year from 2009 to 2020 (see line 658) and we have analysed all data combined for having a whole comprehension and to try to identify some pattern (see lines 635-657).

**4) Discussion:**

**I am not convinced that the data used here allows you to estimate "the volume of bystanders in cases of IPVAW in Spain". In the case of the early studies, to the best of my knowledge, only a dichotomous question is used, so a person answering positively could be aware of one or multiple cases of IPVAW. In the case of the macro-surveys, they only account for cases in which the victims actively reported the cases to other people, but fail to account for other sources by which people could be aware of such cases (e.g., neighbours watching or hearing violent events). I think, at best, you have an estimate that underrepresents actual awareness, but I don't think this is properly discussed or addressed in your article.**

**Author’s response:** According to the reviewer suggestions, we have included a reflection about the limitations of survey data analysed (see lines 696-703).

**When discussing awareness differences across age groups, you attribute them to generational effects ("the data obtained underscore the existence of a certain generational effect"). However, I don't think the data used here allow you to disentangle ageing, period, and cohort effects. I encourage you to revise the wording to ensure that the interpretation is fully consistent with the analytical approach and the data used.**

**Author’s response:** According to the reviewer suggestions, we have avoided the use of the term “generational effect” and pointed out that it could be an “age effect” (see line 716).

**In addition to this, I think some limitations of the study are not appropriately discussed. For example, some of the studies are quite old (>10 years) and attitudes, perceptions and behaviours might have changed since. I would also like to see further discussion of the implications of the study (how can these findings inform future research and practice?, how can we better measure this going forward?, what strategies can be used to mitigate social desirability biases in survey research?).**

**Author’s response:** According to the reviewer suggestions, we have included a reflection about the limitations of survey data analysed related to the changes over the time (see lines 675-696).

In fact, at the beginning of the discussion we present the estimates on the volume of bystanders in Spain across last years (including the results of this paper). Certainly, some of the surveys analyzed are old, but the results they provide offer more complete information on the subject. Moreover, the 4 sociological surveys provide additional valuable information on the (hypothetical) responses of witnesses in IPVAW cases, which offer us the most important result of this work: there is a significant gap between what witnesses say they will do and what they actually do (as shown by the data on complaints). Certainly, social desirability is one of the problems we encounter, but not the only one. We understand that the main result of this paper is precisely to highlight these contradictions between what is said will be done and what is actually done and stated some of the possible explanations, opening the way for its in-depth study in future works.

**Referee 3:**

**Comment 1. In the introduction section, I suggest expanding the literature on the different helping reactions to IPVAW (e.g., informal helping reactions such as "emotional support to the victim" or formal helping responses such as “report to the police”).**

**Author’s response:** According to the reviewer suggestion, we have added some more explanations and literature on the different helping reactions to IPVAW (see lines 173-193).

**Comment 2. On page 9, related to the variables, give an example of each measure, for example, the knowledge of IPVAW and the type of helping behavior.**

**Author’s response**: As the reviewer 2 suggests, we have included a new table (Table 2, see line 309) with all this information about each of the variables analysed (the variable, the question, and the question format).

**Comment 3. In the discussion section, I suggest highlighting the recommendations that intervention programs should be incorporated to improve the bystander's reactions and contribute to developing helpful and efficient active responses.**

**Author’s response:** According to the reviewer suggestions, we have included a reflection about challenges for future bystander intervention programs highlighted by our results (see lines 722-727).
