# Supplementary material for: Bystanders of intimate partner violence against women and their willingness to intervene: An analysis of secondary data in Spain (2005–2020)
Source: PLoS One. 2022 Sep 20;17(9):e0274822. doi: 10.1371/journal.pone.0274822 (PMC9488790; doi:10.1371/journal.pone.0274822)
Supplement: Attachment — Submitted filename: Response_to_Reviewers.docx [file pone.0274822.s002.docx]

Dear editor,

Thanks to you and to the reviewer 1 and the reviewer 3 for the positive feedback about our revised paper (PONE-D-22-02976R1).

According to you and Reviewers 2 suggestions, we have revised and changed our paper “Bystanders of intimate partner violence against women and their willingness to intervene: an analysis of secondary data in Spain (2005-2020)" (PONE-D-22-02976R1) in order to improve it. Specifically we have made the following changes:

**Additional Editor Comments**

**The paper has improved considerably, as pointed out by the reviewers. Authors have taken into account most of the reviewers’ comments to improve the article. Therefore, the paper is close to being eligible for publication on PlosOne.**

**Authors' reply:** Thank you for the positive feedback.

**There are a number of issues that need to be clarified or corrected in the paper. Reviewer 2 points out several errors, methodological and conceptual issues that must be reviewed and modified. If they are taken into account and incorporated in the last version of the manuscript, it will be suitable for publication.**

**In summary, a major revision must be carried out taking into account the reviewer’s comments.**

**Authors' reply:** We have taken all the Reviewers 2 suggestions into account and have carried out a major revision of the paper.

We respond to each of them hereafter. All these changes are marked using the tool “track changes” option in Microsoft Word

**Referees requirements:**

**Referee 2:**

**In general:**

**Throughout the manuscript, there continues to be issues with readability and clarity. I encourage the authors to proofread the paper to fix typos and improve readability.**

**Authors' reply:** We have carefully revised the text of the article to improve its clarity and readability and to eliminate typographical errors. All changes made are marked in the revised document.

**Although the article´s goal is “to estimate the volume of bystanders in cases of IPVAW in Spain between 2005 and 2020”, I continue to believe that this is not achieved. For this, one would ideally conduct a meta-analysis, pooling data from multiple studies (not only publicly available data sets, but also peer-reviewed articles and grey literature). I think being more precise on what the study achieves would provide clarity to the manuscript.**

**Authors' reply:** For improving the clarity about the study achieves, the summary (lines 29-30) and introduction (line 106) have been modified to describe accurately the main aim of our paper.

We thank the reviewer for the idea of doing a meta-analysis to include all the available literature and research and thus reach a more accurate and detailed estimate of the bystanders’ volume in Spain. In fact, we have included this suggestion as a line of future work in the conclusions of our paper (lines 642-645).

**Abstract & Introduction:**

**In the abstract, and introduction, when justifying the period of time covered on the study, it is indicated that it spans from 2005 to the present. Because the sources only cover 2005-2020 (rather than 2022), I would suggest revising the wording for accuracy.**

**Authors' reply:** For improving the accuracy, the summary (line 31) and introduction (line 107) have been modified to match the period of the study conducted (which covers 2005 to 2020, not to the present).

**I think at times the conclusions go beyond the findings. For example, in the abstract it is said that people close to the victims know of their victimisation, and that “although these bystanders claim they would engage in an active and supportive response, this is in fact not always the case.” The wording makes it sound like there is information from the same individuals on both fronts (their awareness of cases of IPV and their willingness to intervene). However, these are different samples and studies, and I think these type of generalisations should be tempered.**

**Authors' reply:** The description of the results in the summary has been modified and tempered in order to avoid possible generalisations, because effectively they are not the same samples or the same studies (see changes in lines 35-39).

**Results:**

**On Table 2 it is indicated that the first three surveys were conducted using face-to-face personal interviews, while the most recent Macro-encuesta used CAPI (which is also a form of personal interviewing). Did the first three studies used PAPI? I would encourage the authors the specify this, and to also include the website where the data can be downloaded, so that readers have all relevant information.**

**Authors' reply:** On Table 1 (Information about each sociological survey included) we have included the information about mode of data collection exactly as appears in technical sheet of each analysed survey: in the first three surveys the technical sheet says that they were conducted using face-to-face interviews during a home visit, and in the last one the technical sheet says that they was conducted by Computer-assisted personal interviews (CAPI).

Additionally we have included on the Table 1 the website where the data can be downloaded, as the reviewer suggests (see line 149).

**In the “Data analysis” section, it is indicated that histograms were used, but they seem inappropriate given that all variables are nominal.**

**Authors' reply:** The error has been corrected by replacing histogram with bar chart (line 180).

**In some of the figures (Figure 2, Figure 6…), the legend (women/men) seems to be missing.**

**Authors' reply:** We have added the legend to the Figures 2 and 6.

**At times the findings are quite repetitive, as the results are disaggregated by sex, then by age, and then by sex and age (both). Since the results are consistent, I would move some of this information to the appendix (e.g., Figures 4 and 5, Figures 8 and 9) to avoid repetition and streamline this section (this would also help reduce the still high number of figures and tables in the main text).**

**Authors' reply:** The Figures mentioned by the reviewer refer to the responses that adult men (FIG4), adult women (FIG5), young men (FIG8) and young women (FIG9) probably would give to IPVAW. This is a very relevant information for the objectives of our study and whose analysis is presented in the text and completed with the figures mentioned.

We understand that if this relevant information is kept as text, but the Figures are moved to the appendix (as suggested by the reviewer) the text does not become clearer if not more confusing.

However, if the editor considers it convenient, we have no objection to move Figures 4, 5, 8 and 9 to an appendix. We await your instructions in this regard.

**Also note that, throughout the paper, you refer to “sex” but the categories that you use (women, men) are characteristic of gender identity rather than sex (usually male female). You might want to review this to ensure internal coherence and consistency with the literature.**

**Authors' reply:** We have revised the Figures and we have changed the legend women – men by female – male to be consistent with the use of the category sex.

**Table 3, as currently drafted, can be misleading. Although the n reported in the table title is 1,579, the percentages reported in rows 2-7 are based on those who were aware, which is a much smaller number (n=864). Please, review this to avoid confusion.**

**Authors' reply:** Effectively, we have corrected the n because data in Table 3 are not related to 1,579 women.

Regarding the macro-survey conducted in 2014, among the women interviewed who had been in an intimate partner relationship at some point in their life (n = 9,807), 16.1% (n = 1,579) had been victim of physical violence and/or sexual violence or had been afraid of their partner or ex-partner. And 81% of the women who had suffered some form of IPVAW (n = 1,279) had explained their situation to another person. Table 3 refers to these 1,279 women, and we have changed the n value in this sense (sse line 355 and line 360).

**I would also suggest revising Tables 3 and 4 (which show very similar content) so that they are presented in the same way (the column headings in Table 3 are row headings in Table 4 at the moment, making the comparison between 2014 and 2019 data difficult).**

**Authors' reply:** To facilitate comparison between Tables 3 and 4, the Table 3 format has been modified (swapping rows and columns), as suggested by the reviewer.

**The authors classify responses encouraging the victim to remain with the perpetrator as “passive”. I understand that they are not active in a positive sense (advising to leave), but they are, indeed, active in a negative sense. For me, inaction or passivity would mean not doing anything, whereas encouragement to remain in the relationship would represent a negative active reaction. Perhaps some additional clarity on why the authors interpreted this as inaction would be helpful here.**

**Authors' reply:** In the introduction (lines 56 to 61) a description was given of what the literature on the subject considers as active/passive responses of bystanders:

"As in the case of other emergency situations, bystanders of VAW and IPVAW have the option of being either “passive” or “inactive”, meaning they may choose not to become involved, to ignore the situation and/or keep quiet and do nothing, supporting the perpetrator and/or blaming the victim, or “active”, meaning to engage and intervene to help the victim providing different forms of informal support to the them (for example, offering assistance, helping them to make decisions, talking to the them, helping or accompanying her to access support services, or helping her to report the case to the police) and/or stop the violence (for example, taking personal action, confronting the aggressor, requesting legal intervention, or reporting the case to the police ) [10-18], thus contributing, or not, to the prevention of such acts of abuse".

In accordance with this criterion, in the analysis of macrosurveys' results (lines 367 to 371), responses that involve doing nothing, as well as those that involve supporting the perpetrator (i.e., showing indifference or encouraging the woman to give the abuser another chance), have been considered as inactive/passive.

**On page 36, when reporting estimates of IPVAW from the most recent Macro encuesta, the authors disaggregate the results by women who had previously had a partner and those who currently had it. However, for the latter, they report prevalence estimates for partners and ex-partners. Is the inclusion of ex-partner correct in this context? (e.g., Among women who had a partner at the time of the 381 interview (n = 6,506), 14.7% (n = 958) had suffered physical, sexual and/or psychological violence at the hands of their partner or ex-partner in the 12 months prior to the interview, 2.9% physical and/or sexual violence (n = 191), and 14.5% psychological violence (n = 943).).**

**Authors' reply:** In order to describe these results, we have followed exactly the criteria established by the Centro de Investigaciones Sociologicas to describe the macrosurveys results obtained.

In fact, in lines 381-388 we have presented two types of results:

First, they are results related to the women interviewed who had previously had a partner:

“Among the women interviewed who had previously had a partner (n = 9,218), 33.6% (n = 3,098) suffered physical, sexual and/or psychological (emotional, controlling, financial or fear-inducing violence) abuse at the hands of their partner or ex-partner at some point in their life; 14.8% experienced physical and/or sexual (n = 1,362) violence; and 33.2% suffered psychological trauma (n = 3,056)”.

Secondly, they are results related to the women interviewed who had a partner at the time of the interview:

“Among women who had a partner at the time of the interview (n = 6,506), 14.7% (n = 958) had suffered physical, sexual and/or psychological violence at the hands of their partner or ex-partner in the 12 months prior to the interview, 2.9% physical and/or sexual violence (n = 191), and 14.5% psychological violence (n = 943)”.

In each case, we have presented results related to the former and the present partners (as in the originals reports of Centro de Investigaciones Sociologicas and Gender Violence Government Delegation).

**Similarly, on page 37, the number of women who reported physical, sexual or emotional violence or fear of their current partner is said to be 592, but in the next paragraph, when referring to the same group, the reported n is 520. Please, carefully review the manuscript to resolve inconsistencies.**

**Author’s reply:** Indeed, the wording of the sentence was misleading and confusing. The sentence has been revised to read as follows:

Lines 593 – 596: Among all women who had suffered physical, sexual or emotional violence or had feared their partner (n = 2,395), 21.7% (n = 520) had reported these aggressions either to the police or directly to the courts (25% in the case of former partners; 5.4% in the case of current partners)

**In Table 5, is the 89.8% for 2013 (victim) correct? Or should this be 8.9%?**

**Authors' reply:** We have corrected the mistake. The data should be 8.9%.

**Discussion:**

**When discussing why bystander responses in this study differ from other studies, the authors might want to mention that this is highly dependant on the responses provided in the surveys. While other studies have offered a wider variety of responses, potentially leading to more selections, the surveys used in this study only included three active responses.**

**Authors' reply:** Thank you for the suggestion that has been included in the discussion (lines 559-561 and 625-626).

**Another limitation that should be noted is the lack of control for potential confounders in the analysis (e.g., the relationship between age or sex and the variables of interest can be moderated by variables such as level of education, and this is something that the analytical approach cannot tell).**

**Authors' reply:** Thank you for the suggestion that has been included in the discussion (lines 630-633).

**Finally, I still feel that the discussion can be strengthen by more clearly stating the contribution of the article and its implications for both research and practice (e.g., the authors mention the limitations of the current variables, but it would be helpful to reflect on the ways forward).**

**Authors' reply**: Thank you for the suggestion that has been included in the discussion (lines 642-645), adding like this some more information to that already included in this regard (lines 646-654).
